# Fresh pomegranate juices from cultivars and local ecotypes grown in southeastern Italy: comparison of physicochemical properties, antioxidant activity and bioactive compounds

**DOI:** 10.1002/jsfa.11456

**Published:** 2021-08-11

**Authors:** Annalisa Tarantino, Graziana Difonzo, Grazia Disciglio, Laura Frabboni, Vito Michele Paradiso, Giuseppe Gambacorta, Francesco Caponio

**Affiliations:** ^1^ Department of Agriculture, Food, Natural Resources and Engineering (DAFNE) University of Foggia Foggia Italy; ^2^ Department of Soil, Plant and Food Science (DISSPA) University of Bari Aldo Moro Bari Italy; ^3^ Department of Biological and Environmental Sciences and Technologies University of Salento Lecce Italy

**Keywords:** pomegranate genotypes, juice quality, physicochemical properties, antioxidant activity, polyphenols, anthocyanins

## Abstract

**BACKGROUND:**

Pomegranate juice has gained attention for its health properties, becoming consequently a highly demanded product. The revival of the pomegranate in Italy, as in other Mediterranean countries, starts with the planting of new intensive orchards characterized by both the new cultivation technique and new varieties. As a result of growing demand and high productivity, pomegranate could become an interesting crop to diversify farm income. This study seeks to determine the aril juice quality attributes and bioactive compounds of six pomegranate cultivars (‘Mollar’, ‘Dente di cavallo’, ‘Acco’, ‘Jolly red’, ‘Wonderful’ and ‘Wonderful Super’) and two local ecotypes (‘Eco BA’ and ‘Eco FG’) grown in Apulia region, southern Italy.

**RESULTS:**

The aril juices were evaluated for their main physicochemical properties (yield, color, pH, total soluble solids content, titratable acidity, sugar–acid ratio), chemical and bioactive compounds (vitamin C, phenolics, anthocyanins and antioxidant activities). ‘Eco BA’, ‘Mollar’ and ‘Jolly red’ genotypes were characterized by the highest maturity index, and then could be considered to be sweet–sour in taste. Total phenols and antioxidant activity were higher in ‘Dente di cavallo’ and ‘Eco FG’ genotypes. ‘Eco FG’ was also the richest in vitamin C, punicalagin and ellagic acids, while ‘Dente di cavallo’, ‘Acco’ and ‘Wonderful’ showed the highest content of the detected anthocyanin compounds.

**CONCLUSION:**

These results contribute to current knowledge about chemical composition, phenolic contents, anthocyanin profiles and antioxidant activity of pomegranate juice from different genotypes, showing in most cases an appreciable juice quality and bioactive profile, although significant differences among them were detected. © 2021 The Authors. *Journal of The Science of Food and Agriculture* published by John Wiley & Sons Ltd on behalf of Society of Chemical Industry.

## INTRODUCTION

Pomegranate (*Punica granatum* L.) is a temperate climate species, mainly cultivated in the Mediterranean area, Southern Asia and several countries of North and South America. Pomegranate for fresh market and consumption is limited due to difficulties in peeling to obtain the arils, and usually they are destined for industrial use.^1^ Today, however, technologies for the separation and mechanical extraction of the grains have become available that reach high levels of efficiency, which can insert pomegranate into the processing lines as a fourth range product either alone or in combination with other fruit and vegetables. The fruit is used extensively in food and beverage industries for fortification and formulation of food products including yogurt, beverages, jellies, jams, cereal bars, nutraceutical, pharmaceutical and cosmeceutical products,^2^ due to the increasing interest towards dietary sources of phytochemicals. In this regard, pomegranate juice has shown an antioxidant activity three times greater than that of red wine or green tea,^3^ and two, six and eight times higher than the activity detected, respectively, in grapes, blueberries, grapefruit and orange juice.^4^ Pomegranate fruit is rich in bioactive compounds such as phenolic acids, flavonoids (anthocyanins, proanthocyanidins, ellagitannins and gallotannins), vitamins, organic acids (ascorbic, citric and malic), sugars (fructose and glucose), natural antioxidants and mineral elements.^5^, ^6^ As for the taste and flavor of pomegranate, the trait highly appreciated by consumers is the sugar–acid ratio.^7^ The aforementioned attributes of the fruits differ depending on the cultivar, location and climate conditions, degree of ripeness, method of cultivation and storage. Moreover, in the case of pomegranate juices these are also related to the production method, as juice may be produced only from sarcotestas or from whole fruits.^8^, ^9^, ^10^

A vast range of pomegranate varieties can be found in Italy. It is very difficult to establish their number, both because of the occurrence of numerous synonyms, derived from the tendency to identify a variety with the name of the location or country of origin, and because of the convenience to group them by organoleptic characteristics (juice flavor), of arils (color) and coloring of the fruit.^11^ In Italy, pomegranate is cultivated in southern semiarid areas such as Sicily, Apulia, Basilicata and Calabria regions. Particularly in Apulia region, over the last decade the area used for pomegranate cultivation has increased significantly, from 8 ha in 2009 to about 1000 ha (60% of the total national) currently cultivated.^12^, ^13^ The most widespread commercial cultivars currently grown in Italy are ‘Wonderful’ (originating from Florida), ‘Acco’ (from Israel) and ‘Dente di cavallo’ (from Italy), but many other interesting foreign cultivars, including ‘Mollar’ (from Spain), ‘Jolly red’, seedless (from Israel) and numerous local ecotypes, among which ‘EcoBA’ and ‘EcoFG’, tested for the first time in this research, could be introduced into Italian crops. In this regard, little is known about the different characteristics of varieties despite recent findings indicating a broad genetic and geographic diversity. Considering the growing interest in the production and processing of pomegranate, the need for scientific studies on the quality of the product have thus been stimulated. Following this, the aim of this study was to evaluate and compare in a semiarid environment of Apulia region of Italy, characterized also by a high wind speed, the physicochemical characteristics (color, pH, acidity, total soluble solids), antioxidant activity, and chemical and bioactive contents (vitamin C, phenolic content, anthocyanins) of juice in the above‐mentioned genotypes, in order to provide information relevant to assist in cultivar selection for food and industrial purposes.

## MATERIALS AND METHODS

### Site and fruit collection

The study was undertaken during the 2018 season to evaluate the physicochemical traits, antioxidant activity and bioactive compounds of the juices of six commercial cultivars (‘Mollar’,’Dente di cavallo’, ‘Acco’, ‘Jolly red’, ‘Wonderful’ and ‘Wonderful Super’) and two local ecotypes (‘EcoBA’ and ‘EcoFG’), hereafter all referred to as genotypes. They were grown for comparison in the same private orchard in Foggia (Apulia region, southern Italy: 41° 27′ 08″ N, 15° 31′ 56″ E; altitude 54 m above sea level). The site of the research was in a typical semiarid zone, characterized by a Mediterranean climate, with a mild winter and dry, warm summer. The climate is classified as an ‘accentuated thermo‐Mediterranean’ (UNESCO–FAO classification), with a temperature that may fall below 0 °C in winter and exceed 40 °C in summer. Rainfall is unevenly distributed throughout the year and is mostly concentrated in the winter months, with a long‐term annual average of 559 mm.^14^ The plants, 3 years old, were spaced at 5.5 × 3.0 m, grown from cuttings and trained to a vase shape. A drip irrigation system was used for fertigation purposes. Fruit harvest was carried out at the mature stage, according to other authors,^15^ at different dates: the early variety, ‘Acco’, at the beginning of October, the medium‐early variety, ‘Mollar’, within the first ten days of October, the other intermediate ones in mid‐October, and the late cultivars ‘Wonderful’ and ‘Wonderful super’ at the end of October. Samples of 12 fruits from each genotype (four per plant) were randomly collected in plastic bags and stored in a portable ice box for transport to the laboratory. Images of the collected fruits from the different genotypes are provided in Appendix S1. Fruits were cut longitudinally and arils, manually separated, were squeezed through a metal sieve to obtain the juice. The juice was filtered and separated into two aliquots, one of which was immediately frozen at −20 °C for analysis by high‐performance liquid chromatography (HPLC). The other aliquot of fresh juice was used for determination of physicochemical parameters.

### Physicochemical analysis of juice

The fruit juice was placed in a colorless glass Petri dish and color was recorded using a chroma meter (CR‐400, Konica Minolta Sensing Inc., Osaka, Japan). In the CIELab color space, L* indicates brightness, a* ranges from green (−a*) to red (+a*), and b* varies from blue (−b*) to yellow (+b*).

Juice yield (cm^3^ 100^−1^ g of arils), sugar content (TSS, °Brix), pH, total acidity and maturity index (MI) as the ratio of °Brix/total acidity were determined. A digital refractometer was used to measure the °Brix value, and a semi‐automatic titrator (PH‐Burette 24, Crison, Spain) was used for total titratable acidity determination. In particular, 5 mL juice was diluted to 50 mL with distilled water and titrated with 0.1 mol L^−1^ NaOH to pH 8.1. Total titratable acidity was calculated as g citric acid 100 mL^−1^ juice.

### Total phenol content (TPC) and antioxidant activity evaluation

TPC was determined by the Folin–Ciocalteu method according to Difonzo *et al*.,^16^ with some modifications. 20 μL juice was added to 980 μL double‐distilled water and 100 μL Folin–Ciocalteu reagent. After 3 min, 5% Na_2_CO_3_ solution was added, following incubation at room temperature for 60 min. The absorbance was read at 720 nm using a Cary 60 spectrophotometer (Agilent, Milan, Italy). TPC was expressed as gallic acid equivalents (GAE) in mg 100 mL^−1^ juice.

The antioxidant activity was evaluated by ABTS (2,2′‐azinobis‐(3‐ethylbenzothiazoline‐6‐sulfonic acid assay)–TEAC (Trolox equivalent antioxidant capacity) assay, and was carried out as described by Re *et al*.,^17^ with modifications reported in Ranieri *et al*.^18^ The results were expressed in mmol Trolox equivalents (TE) 100 mL^−1^ of juice.

### Quantitation of vitamin C

Vitamin C was quantified by HPLC‐UV following a modified procedure according to Aschoff *et al*.^19^ and Difonzo *et al*.^16^ The HPLC analysis was carried out using an Agilent 1260 Infinity HPLC system (Agilent Technologies, Palo Alto, CA, USA). The HPLC system consisted of a 1260 QUAT PUMP quaternary pump, 1260 ALS autosampler, 1260 TCC stationary phase compartment, 1269 DAD VL diode array detector and Openlab software for data acquisition and processing. The HPLC system was operated with a RP‐C18 column Acclaim™ 120 (150 × 3.0 mm, 3 μm particle size, 120 Å pore size; Thermo Scientific). Water containing 1% (w/v) metaphosphoric acid, adjusted to pH 2.6 with 2 mol L^–1^ aqueous K_2_HPO_4_, was used as eluent, applying an isocratic elution at 25 °C. Total run time was 20 min at a flow rate of 1.0 mL min^−1^. Ascorbic acid was detected at a wavelength of 254 nm and quantitated by external linear calibration. The reported vitamin C concentrations equal the sum of ascorbic and dehydroascorbic acid.

### HPLC–diode array detection (DAD) analysis of polyphenols

Polyphenol analyses (anthocyanins and non‐anthocyanin phenolic compounds) were performed according to Fischer *et al*.^20^ and Tarantino *et al*.^21^ using a Dionex HPLC system (Thermo Scientific, Germering, Germany) equipped with a WPS‐3000 RS autosampler, HPG‐3200 RS pump, Jetstream column oven, and L‐2450 diode array detector. The separation was carried out with an analytical column (3 μm particle size, 120 Å pore size, 150 × 3.0 mm; RP‐C18 column Acclaim™ 120, Thermo Scientific) operated at 30 °C. The diode array detector was set at an acquisition range of 200–600 nm. For the quantitation of the detected compounds two external calibration curves were made with ellagic acid and with malvidin‐3‐glucoside to express the results of polyphenols and anthocyanins, respectively.

### Statistical analysis

Analyses of color juice were replicated six times for each genotype, whereas all other physicochemical and bioactive compound analyses were replicated three times for each genotype. The results were evaluated with one‐way analysis of variance (ANOVA) using JMP software (SAS Institute Inc., Cary, NC, USA) and average values were compared with Tukey's test. Standard deviations (SD) were calculated using Excel software of the Office 2007 suite (Microsoft Corporation, Redmond, WA, USA). Percentage values were transformed to arcsine prior to ANOVA in Minitab. Principal component analysis was carried out by means of Minitab 17 (Minitab Inc., State College, PA, USA).

## RESULTS AND DISCUSSION

### Physicochemical properties

As indicated by the color data (Table 1), the lightness parameter (L*) was highest in ‘Eco FG’ (25.6) although not statistically different from Jolly red (24.4), which in turn was not different from ‘Mollar’ (23.5) or ‘Eco BA’ (21.9) but different from the other compared genotypes (ranging between 20.3 and 20.8). The redness index (a*) showed the highest value in ‘Dente di cavallo’ (13.0), not significantly different from either ‘Acco’ (12.6) or Wonderful’ (12.4), but significantly higher than ‘Eco FG’ (11.7), which in turn were statistically higher than both ‘Wonderful super’ (8.7) and ‘Jolly Red’ (8.3). The lowest significantly data were obtained from ‘Eco BA’ (4.9) and ‘Mollar’ (4.1). The yellowness index (b*) showed the highest value in ‘Eco FG’ (5.9), although not significantly different from ‘Jolly Red’ (3.3) or ‘Eco BA’ (2.1), which in turn were statistically different from the remaining genotypes (ranging from 0.2 to 1.0).

**Table 1:** Color characteristics of pomegranate juice from different genotypes

Genotype	Color parameter		
	L*	a*	b*
Mollar	23.5 ± 1.5b	4.1 ± 1.0d	1.0 ± 0.6b
Dente di cavallo	20.3 ± 0.3d	13.0 ± 0.8a	0.2 ± 0.1c
Acco	20.8 ± 0.5d	12.6 ± 0.6ab	0.2 ± 0.1c
Jolly red	24.4 ± 2.5ab	8.3 ± 0.5c	3.3 ± 2.7a
Wonderful	20.5 ± 0.2d	12.4 ± 0.4ab	0.2 ± 0.1c
Wonderful super	20.5 ± 0.7d	8.7 ± 0.2c	0.3 ± 0.1c
EcoBA	21.9 ± 0.4bc	4.9 ± 0.6d	2.1 ± 0.6a
EcoFG	25.6 ± 1.6a	11.7 ± 0.9b	2.9 ± 0.5a

Values are the mean of six replications (± standard deviations). Values followed by the same letter within the same column are not statistically different at *P* < 0.05 determined by ANOVA and Tukey's test.

While our data of juice color attributes were consistent with those of Ferrara *et al*.,^22^ those for ‘Acco’ and ‘Wonderful’ were lower than the same cultivars described by Passafiume *et al*.^23^

Other physicochemical parameters affecting pomegranate quality, namely juice yield, pH, TSS, TA, and TSS/TA, are reported in Table 2. One of the most important parameters from an industrial point of view is the juice yield, obtained from 100 g arils, which in this study was significantly affected by genotype. ‘Wonderful super’ produced the greatest value (88.9 cm^3^ 100 g^−1^), although not statistically different from ‘Jolly red’, ‘Acco’, ‘Eco BA’ or ‘Mollar’ (values ranging between 72.5 and 85.6 cm^3^ 100 g^−1^), but was significantly different from the remaining genotypes (ranging between 72.3 and 78.3 cm^3^ 100 g^−1^). The average juice yield of genotypes analyzed was very similar to that reported by Ferrara *et al*.^12^ Moreover, our data for ‘Acco’ (85.5 cm^3^ 100 g^−1^) and ‘Wonderful’ (78.3 cm^3^ 100 g^−1^) were higher than those of Passafiume *et al*.,^23^ which presented values of juice yield of 67.3 and 38.4 cm^3^ 100 g^−1^, respectively.

**Table 2:** Physicochemical characteristics of juice from the different pomegranate genotypes

Genotype	Juice yield (cm^3^ 100^−1^ g arils)	TSS(°Brix)	pH	TA (g citric acid 100 mL^−1^)	TSS/TA (°Brix/% citric acid)
Mollar	78.5 ± 13.4ab	14.7 ± 0.3c	3.0 ± 0.1bc	0.6 ± 0.1d	26.0 ± 4.2ab
Dente di cavallo	72.5 ± 10.8b	16.8 ± 0.2ab	2.8 ± 0.1c	1.6 ± 0.1b	10.8 ± 0.5d
Acco	85.5 ± 6.4a	16.2 ± 0.1b	2.8 ± 0.1c	1.7 ± 0.2b	9.4 ± 0.9d
Jolly red	85.6 ± 4.8a	14.7 ± 0.4c	3.6 ± 0.3a	0.6 ± 0.1d	23.4 ± 1.4b
Wonderful	78.3 ± 3.4b	17.7 ± 0.2a	3.3 ± 0.1b	2.3 ± 0.2a	7.8 ± 1.0e
Wonderful super	88.9 ± 2.1a	16.9 ± 0.2ab	2.8 ± 0.1c	2.2 ± 0.2a	7.8 ± 0.8e
Eco BA	84.0 ± 5.7a	17.0 ± 0.9a	3.6 ± 0.1a	0.6 ± 0.1d	27.6 ± 1.9a
Eco FG	76.1 ± 2.0b	17.8 ± 0.9a	3.5 ± 0.1a	1.3 ± 0.1c	14.7 ± 0.7c

Values are the mean of three replications (± standard deviations). Values followed by the same letter within the same column are not statistically different at *P* < 0.05 determined by ANOVA and Tukey's test.

Concerning TSS, most of the genotypes had average data between 16.2 and 17.8 °Brix, which were significantly higher than those of ‘Jolly red’ and ‘Mollar’ (14.7 °Brix), although the latter was higher than the minimum (12 °Brix) required for commercial use.^24^ The average °Brix values of genotypes analyzed in this research were very similar to those reported in other studies,^12^, ^23^, ^25^, ^26^ but higher than the values reported by Martínez *et al*.^7^ As for ‘Dente di cavallo’ and ‘Wonderful’, our data were slightly lower than those of the same cultivars studied by Adiletta *et al*.^27^

The highest pH values were obtained in ‘Jolly red’, ‘Eco BA’ and ‘Eco FG’, which were statistically different from ‘Wonderful’ (3.3), which in turn was higher than the remaining genotypes (values between 2.8 and 3.0). Overall, our data are in a similar range to those obtained from previous studies,^2^, ^7^, ^12^ but lower than those obtained by Fernandes *et al*.,^26^ and Adiletta *et al*.^27^

The TA values showed a wide range (from 0.6 to 2.2% citric acid). The highest values were found in ‘Wonderful’ (2.3%) and ‘Wonderful Super’ (2.2%), significantly greater than ‘Acco’ (1.7%) and ‘Dente di cavallo’ (1.6%) and both statistically higher than ‘Eco FG’ (1.3%). The lowest value (0.6%) was observed in ‘Eco BA’ and ‘Molar’ genotypes. In agreement with Zarei *et al*.^28^ genotypes with higher acid content showed a correspondingly lower pH. Our value for ‘Wonderful’ (2.3%) is higher than those obtained in other research,^26^, ^27^ in which the TA values ranged between 1.14% and 1.68%. Furthermore, our data for ‘Dente di cavallo’ (1.6%) and for ‘Mollar’ (0.60%) were, respectively, higher than those of Adiletta *et al*.^27^ (0.41%) and Fernandes *et al*.^26^ (0.32%).

The TSS/TA ratio, indicative of maturation index (MI), was highest in ‘Eco BA’ (27.6), ‘Mollar’ (26.0), and ‘Jolly red’ (23.4) genotypes which, according to the ranking established for Spanish cultivars,^7^, ^29^ could be considered sour–sweet to sweet. Intermediate values were shown for ‘Eco FG’ (14.7), ‘Dente di cavallo’ (10.8) and ‘Acco’ (9.4), which could be considered ‘sour–sweet’, whereas the lowest value (7.8) was detected for both ‘Wonderful’ and ‘Wonderful super’ genotypes, which could be considered sour. Moreover, Chace *et al*.^30^ have reported that pomegranates are suitable for the fresh market when their acidity content is lower than 1.8% and their MI is between 7 and 12. According to these criteria, the cultivars ‘Wonderful’ and ‘Wonderful super’ analyzed in this study were not suitable for fresh consumption.

### Bioactive profile

#### Total phenols, antioxidant activity and vitamin C

**Figure 1:**
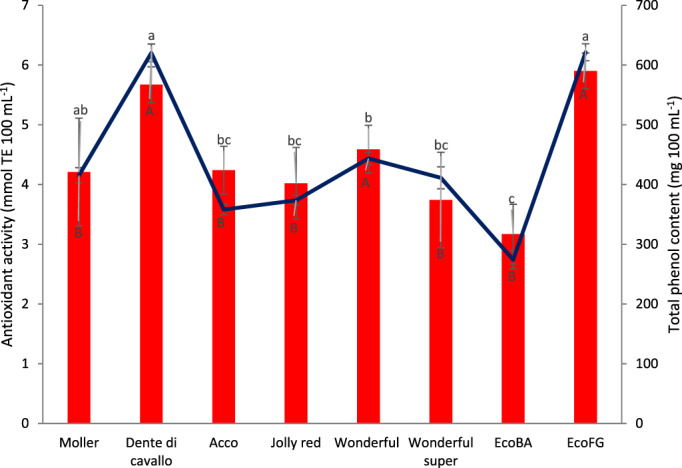
Antioxidant activity (line) and total phenol content (bars) of juice from pomegranate genotypes. Different letters (upper‐ and lower‐case letters refer to total phenol content and antioxidant activity, respectively) indicate statistical differences at *P* < 0.05.

The total phenol content (TPC) and antioxidant activity of the pomegranate juices are reported in Fig. 1. The TPC was significantly higher in ‘Dente di cavallo’, ‘Wonderful’ and ‘Eco FG’, whereas no significant differences were found among remaining genotypes. The TPC range of these genotypes was similar to that reported for some cultivars grown in southern Turkey and higher than Greek accessions and Chilean genotypes.^31^

Concerning antioxidant activity, the studied genotypes showed a variability according to their total phenol content. In fact, genotypes with the highest TPC values also had higher antioxidant activity than those characterized by lowest TPC. In particular, the highest values were found in ‘Dente di cavallo’ and ‘Eco FG’, followed by ‘Mollar’, which in turn was not significantly different from the other genotypes except for ‘Eco BA’ that was characterized by the lowest value.

Pomegranate has been promulgated as a polyphenol‐rich food with health beneficial effects due to its high antioxidant capacity; thus is commonly referred to as a ‘superfruit’. Seeram *et al*.^32^ compared the antioxidant capacity of different polyphenol‐rich beverages, such as different kinds of juices and tea as well as red wine. Among these beverages, pomegranate juice was found to exhibit the highest TEAC (Trolox equivalents antioxidant capacity) value (42 mmol L^−1^). However, the *in vitro* antioxidant capacity may not correlate with antioxidant effects in humans, since the bioavailability and bioconversion are not taken into consideration. Nevertheless, in several studies pomegranate juice has been demonstrated to exert anti‐inflammatory effects in humans.^20^

**Figure 2:**
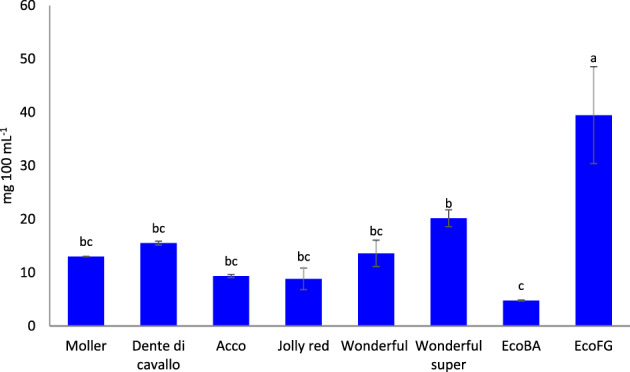
Vitamin C of pomegranate juice from different genotypes. Different letters indicate statistical differences at *P* < 0.05.

Moreover, the content of vitamin C was evaluated since it represents one of the most important antioxidant molecules in pomegranate juice. According to the results of antioxidant activity, ‘Eco FG’ showed the highest content of vitamin C (39.5 mg mL^−1^), whereas ‘Eco BA’ had the lowest (4.8 mg 100 mL^−1^) (Fig. 2). Ferrara *et al*.^12^ found a similar range of vitamin C content in different Apulian genotypes.

#### Anthocyanins and non‐anthocyanin phenolic compounds by HPLC‐DAD

Anthocyanins, naturally occurring colorants of the flavonoid family, are a class of secondary metabolites responsible for the purple, blue, red and orange colors of several fruits and different plants. Fruit exterior color is an important characteristic with regard to the commercial value and aesthetic quality of the fruit. There is a large variability in pomegranate fruit color, ranging from white to red to dark red. It is hypothesized that the color is due to anthocyanin composition and change in their proportion during fruit maturation, and pH value. The six most common anthocyanidins found in fruits and vegetables are cyanidin, pelargonidin, malvidin, delphinidin, petunidin and peonidin.^33^ Usually, the anthocyanidins are linked to different sugars, which confer stability and water solubility to the molecule. In addition to its colorant properties, anthocyanins have been associated with a wide range of biological, pharmacological, anti‐inflammatory, antioxidative and chemoprotective properties. Their potential health benefits are provoking an increasing interest in these compounds.^34^ Analysis of the anthocyanin content showed how the genotypes were characterized by their own profile (Table 3). The highest content of delphinidin‐3,5‐diglucoside was found in ‘Dente di cavallo’, followed by ‘Wonderful’ and ‘Acco’; this latter was characterized by the highest levels of delphinidin‐3‐glucoside and cyanidin‐rutinoside. ‘Wonderful’ was characterized by the highest level of cyanidin‐3,5‐diglucoside, followed by ‘Dente di cavallo’ and ‘Acco’. Overall, ‘Dente di cavallo’, ‘Acco’ and ‘Wonderful’ showed the highest content of the detected anthocyanin compounds, according to the results of the determination of the color parameter a*.

**Table 3:** Anthocyanin profile of pomegranate juice from different genotypes

Genotype	*Del‐3*,*5‐diglc*	*Cya‐3*,*5‐diglc*	*Del‐3‐glc*	*Cya‐rut*
Mollar	93.1 ± 3.8f	77.8 ± 5.24f	112.5 ± 4.1d	180.3 ± 1.6e
Dente di cavallo	957.3 ± 14.4a	530.6 ± 26.6ab	716.0 ± 28.1c	493.7 ± 44.8c
Acco	621.9 ± 29.9c	495.7 ± 25.7bc	876.7 ± 12.3a	942.6 ± 39.0a
Jolly red	98.4 ± 0.9f	99.9 ± 3.6f	126.4 ± 26.9c	215.2 ± 44.6de
Wonderful	775.4 ± 4.1b	543.9 ± 31.3a	785.5 ± 12.6b	741.7 ± 16.0b
Wonderful super	438.1 ± 4.3d	427.0 ± 5.9cd	670.8 ± 2.1c	890.6 ± 34.6a
Eco BA	94.8 ± 6.3f	190.0 ± 32.8e	55.7 ± 5.5e	308.5 ± 57.2d
Eco FG	199.9 ± 54.8e	373.8 ± 49.7d	172.4 ± 4.9d	296.1 ± 23.5d

Means ± standard deviations. The results are expressed as mg L^−1^ malvidin‐3‐glucoside. Values followed by the same letter within the same column are not statistically different at *P* < 0.05 determined by ANOVA and Tukey's test.

Cya, cyanidin; Del, delphinidin; glc, glucoside; rut, rutinoside.

With regard to non‐anthocyanin phenolic compounds, pedunculagin and punicalagin were the most abundant compounds in all genotypes. ‘Eco FG’ had the highest content of punicalagin, ellagic acid, ellagic acid pentoside and deoxyhexoside compared to the other genotypes (Table 4). ‘Dente di cavallo’ had a higher concentration of galloyl hexoside, pedunculagin and casuarinin. Numerous recent scientific publications investigating the health benefits of pomegranate juice showed that the primary cause of the positive effect of pomegranate is linked to the phenolic profile, such as ellagitannins. Among them, punicalagins are reported to possess remarkable anti‐inflammatory and anti‐genotoxic properties, while ellagic acid has been shown to possess antioxidant, anticancer and anti‐atherosclerotic activities. In this framework, ‘Eco FG’ and ‘Dente di cavallo’ seem to be the most promising from a bioactive and health‐related point of view.^35^, ^36^

**Table 4:** Phenolic profile of pomegranate juice from different genotypes

Genotype	*Galloyl‐hex*	*Pedunculagin*	*Punicalagin*	*Casuarinin*	*Ellagic acid‐pent*	*Ellagic acid‐deoxyhex*	*Ellagic acid*
Mollar	35.75 ± 1.81c	121.44 ± 10.31e	255.32 ± 42.37bc	22.64 ± 0.92c	38.07 ± 8.14c	10.35 ± 1.62bc	18.85 ± 5.52b
Dente di cavallo	55.45 ± 0.22a	399.84 ± 13.90a	296.35 ± 5.42b	48.06 ± 0.06a	67.46 ± 4.80ab	13.84 ± 2.73abc	22.53 ± 3.91b
Acco	38.45 ± 0.33c	168.43 ± 5.97d	164.85 ± 12.15de	23.81 ± 0.07c	41.42 ± 0.42bc	9.24 ± 1.08bc	11.22 ± 2.85b
Jolly red	23.04 ± 1.48d	226.58 ± 9.10bc	165.22 ± 0.24de	35.75 ± 2.52b	34.32 ± 2.21c	15.45 ± 0.88ab	22.97 ± 5.31b
Wonderful	50.73 ± 0.95ab	200.21 ± 5.03 cd	237.80 ± 18.95bcd	32.46 ± 1.67b	58.44 ± 5.44ab	10.89 ± 0.61abc	19.08 ± 2.04b
Wonderful super	43.43 ± 1.66bc	188.77 ± 10.93d	193.65 ± 14.90cde	26.91 ± 0.21c	47.92 ± 2.43abc	12.49 ± 1.01abc	14.76 ± 0.46b
Eco BA	20.69 ± 1.37d	32.22 ± 5.14f	146.26 ± 2.91e	14.13 ± 0.01d	30.46 ± 2.52c	6.98 ± 0.35c	16.74 ± 0.24b
Eco FG	49.87 ± 6.64ab	255.03 ± 7.04b	519.37 ± 5.43a	27.89 ± 3.43bc	64.97 ± 11.47a	17.69 ± 3.69a	41.40 ± 4.68a

Means ± standard deviations. The results are expressed as mg L^−1^ ellagic acid. Values followed by the same letter within the same column were not statistically different at *P* < 0.05 determined by ANOVA and Tukey's test.

hex, hexoside; pent, pentoside; deoxyhex, deoxyhexoside.

**Figure 3:**
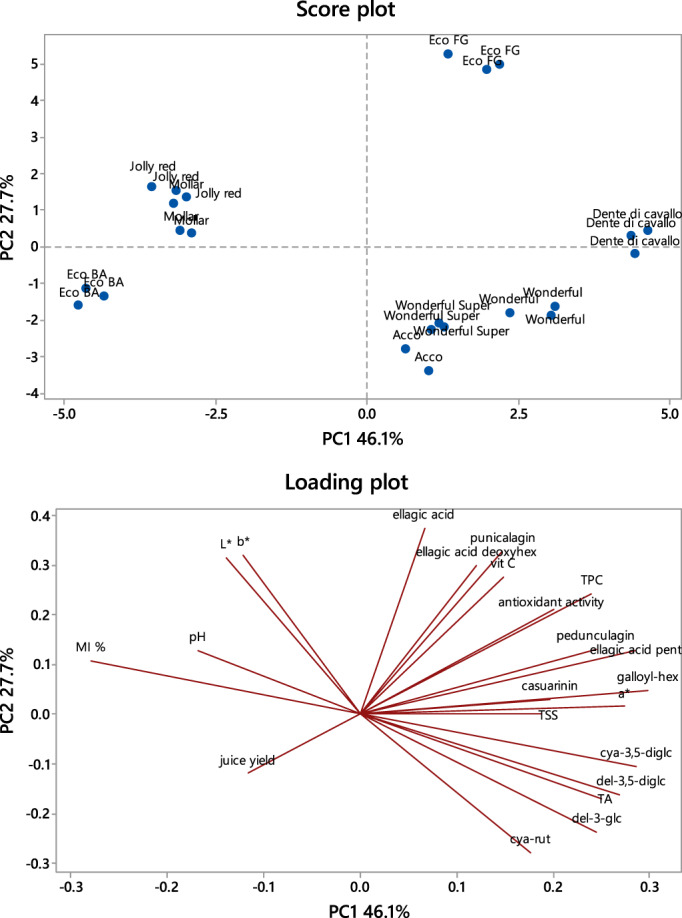
Principal component analysis bi‐plot (PC1 *vs*. PC2), showing the spatial distribution of the pomegranate genotypes (top) and of the whole dataset (bottom).

Analyzing the whole dataset by principal component analysis (PCA) (Fig. 3), the first principal component (PC1) explained more than 40% of the total variability, whereas the second PC (PC2) was responsible of about 30% of the total variability. The combination of datasets resulted in a clear‐cut differentiation of ‘Eco BA’, ‘Jolly red’ and ‘Mollar’ from the other ecotypes on PC1. According to the results discussed previously, the marker parameters that contribute to this differentiation were those related to the bioactive profile, anthocyanin and non‐anthocyanin polyphenols, TSS (positive loadings on PC1) and some physicochemical parameters such as MI, juice yield and pH (negative loading on PC1). PC2 explained the separation between ‘Eco FG’ from ‘Acco’ and ‘Wonderful’ genotypes, especially due to anthocyanin compounds (negative loading on PC2), non‐anthocyanin phenolic compounds and bioactive profile (positive loading on PC2). In fact, ‘Eco FG’ was characterized by high content of TPC, antioxidant activity, vitamin C and non‐anthocyanin phenolic compounds, whereas ‘Acco’, ‘Wonderful’ and ‘Wonderful super’ were characterized by high content of anthocyanins and total acidity.

## CONCLUSIONS

The research was carried out in order to provide information to assist in cultivar selection for food and industrial purposes. A significant variability has been observed for the qualitative and phytochemical traits among the pomegranate genotypes. The ‘Dente di cavallo’, ‘Acco’, ‘Wonderful’ and ‘Eco FG’ genotypes showed the highest values of a* color parameter. The juice yield, the most important parameter from an industrial point of view, was significantly higher in ‘Wonderful super’, ‘Mollar’, ‘Dente di cavallo’, ‘Acco’ and ‘Jolly red’ than in the remaining genotypes. Concerning TSS, all compared genotypes had °Brix values higher than the minimum value (12 °Brix) required for commercial use. The ‘Wonderful super’ and ‘Wonderful’ genotypes were characterized by the significantly highest TA values and for this reason considered sour.

Based on these results, almost all pomegranate genotypes studied are suitable for both the fresh market and processing. Wonderful super’ and ‘Wonderful’ genotypes are more suitable for use in industry.

Considering the bioactive profile, ‘Dente di cavallo’ was one of the most promising cultivars both for anthocyanin content and for phenolic compounds; ‘Eco FG’ was characterized by the highest levels of ellagitannins, ellagic acid and vitamin C. According to these results, both these cultivars also showed the highest values of antioxidant activity determined *in vitro*, confirming that they could be promoted for their high bioactive potential.

These results contribute to current knowledge about chemical composition, phenolic content, anthocyanin profiles and antioxidant activity of pomegranate juice from different genotypes. It is therefore concluded that the promotion of health benefits attributed to pomegranates should be linked to variety and end‐user eating habits.

This information may be useful to producers, breeders and processors. Further studies are needed to improve the knowledge on pomegranate cultivars and ecotypes collected in the local germplasm of different Italian regions, as promising genotypes for either breeding programs or commercial exploitation as fresh or processed fruit.

## CONFLICT OF INTEREST

The authors declare that they have no conflict of interest.

## AUTHOR CONTRIBUTIONS

Tarantino A, Disciglio G and Frabboni L dealt with the conceptualization of the agronomical aspects with the relative physicochemical analyses of juice; Difonzo G, Paradiso VM, Gambacorta G and Caponio F dealt with the conceptualization and relative chemical characterization of bioactive compounds in juices.

## Supporting information


**Appendix S1** Supporting information

## References

[1] Artes F , Tudela JA and Villaescusa R , Thermal postharvest treatments for improving pomegranate quality and shelf life. Postharvest Biol Technol 18:245–251 (2000).

[2] Cassell D , Pomegranate retains superfruit status. Food Process. Available: http://www.foodprocessing.com/articles/2012/pomegranate-retains-superfruit-status/ (2012).

[3] Gil MI , Tomás‐Barberán FA , Hess‐Pierce B , Holcroft DM and Kader AA , Antioxidant activity of pomegranate juice and its relationship with phenolic composition and processing. J Agric Food Chem 48:4581–4589 (2000). 11052704 10.1021/jf000404a

[4] Rosemblat M and Aviram M , Antioxidative properties of pomegranate: in vitro studies, in Pomegranate: Ancient Roots to Modern Medicine (Medicinal and Aromatic Plants‐Industrial Profile), ed. by Heber D , Schulman RN and Seeran NP . CRC Press, Boca Raton. FL (2006).

[5] Wang R , Ding Y , Liu R , Xiang L and Du L , Pomegranate: constituents, bioactivities and pharmacokinetics. Fruit Veg Cereal Sci Biotechnol 4:77–87 (2010).

[6] Li X , Wasila H , Liu L , Yuan T , Gao Z and Zhao B , Physicochemical characteristics, polyphenol compositions and antioxidant potential of pomegranate juices from 10 Chinese cultivars and the environmental factors analysis. Food Chem 175:575–584 (2015). 25577122 10.1016/j.foodchem.2014.12.003

[7] Martínez JJ , Melgarejo P , Legua P , Garcia‐Sanchez F , and Hernández F . Genetic diversity of pomegranate germplasm collection from Spain determined by fruit, seed, leaf and flower characteristics. PeerJ, doi: 10.7717/peerj.2214 (2016), 4, e2214. 27547535 PMC4957998

[8] Legua P , Malgarejo P , Martínez JJ , Martínez R and Henrnández F , Evaluation of Spanish pomegranate juices: organic acids, sugars, and anthocyanins. Int J Food Prop 15:481–494 (2012).

[9] Mditshwa A , Fawole OA , Al‐Said F , Al‐Yahyai R and Opara UL , Phytochemical content, antioxidant capacity and physicochemical properties of pomegranate grown in different microclimates in South Africa. South Afr J Plant Soil 30:81–90 (2013).

[10] Mphahlele RR , Caleb OJ , Fawole OA and Opara UL , Effects of different maturity stages and growing locations on changes in chemical, biochemical and aroma volatile composition of ‘wonderful’ pomegranate juice. J Sci Food Agric 96:1002–1009 (2015). 25809070 10.1002/jsfa.7186

[11] Ferrara G , Mazzeo A , Pacucci C , Pacifico A , Gallo V , Cafagna I *et al*., Melograno: un'opportunità per diversificare il reddito. L'Informatore Agrario 6:52–56 (2014).

[12] Ferrara G , Cavoski I , Pacifico A , Tedone L and Mondelli D , Morpho‐pomological and chemical characterization of pomegranate (*Punica granatum* L.) genotypes in Apulia region, southeastern Italy. Sci Hortic 130:599–606 (2011).

[13] Anonymous . Comunicati Stampa. Available: www.puglia.coldiretti.it. (2019).

[14] Ventrella D , Charfeddine M , Moriondo M , Rinaldi M and Bindi M , Agronomic adaptation strategies under climate change for winter durum wheat and tomato in southern Italy: irrigation and nitrogen fertilization. Reg Environ Change 12:407–412 (2012).

[15] Al‐Maiman SA and Ahmad D , Changes in physical and chemical properties during pomegranate (*Punica granatum* L.) fruit maturation. Food Chem 76:437–441 (2002).

[16] Difonzo G , Vollmer K , Caponio F , Pasqualone A , Carle R and Steingass CB , Characterisation and classification of pineapple (*Ananas comosus* [L.] Merr.) juice from pulp and peel. Food Control 96:260–270 (2019).

[17] Re R , Pellegrini N , Roteggente A , Pannola A , Yang M and Rice‐Evans C , Antioxidant activity applying an improved radicalcation decolorization assay. Free Radical Biol Med 26:1231–1237 (1999). 10381194 10.1016/s0891-5849(98)00315-3

[18] Ranieri M , Di Mise A , Difonzo G , Centrone M , Venneri M , Pellegrino T *et al*., Green olive leaf extract (OLE) provides cytoprotection in renal cells exposed to low doses of cadmium. PLoS One 14:e0214159 (2019). 30897184 10.1371/journal.pone.0214159 PMC6428325

[19] Aschoff JK , Kaufmann S , Kalkan O , Neidhart S , Carle R and Schweiggert RM , In vitro bioaccessibility of carotenoids, flavonoids, and vitamin C from differently processed oranges and orange juices [*Citrus sinensis* (L.) Osbeck]. J Agric Food Chem 63:578–587 (2015). 25539394 10.1021/jf505297t

[20] Fischer UA , Carle R and Kammerer DR , Identification and quantification of phenolic compounds from pomegranate (*Punica granatum* L.) peel, mesocarp, aril and differently produced juices by HPLC‐DAD‐ESI/MS. Food Chem 127:807–821 (2011). 23140740 10.1016/j.foodchem.2010.12.156

[21] Tarantino A , Difonzo G , Lopriore G , Disciglio G , Paradiso VM and Caponio F , Bioactive compounds and quality evaluation of ‘wonderful’ pomegranate fruit and juice as affected by deficit irrigation. J Sci Food Agric 100:5539–5545 (2020). 32596812 10.1002/jsfa.10606

[22] Ferrara G , Giancaspro A , Mazzeo A , Giove SL , Matarrese AM , Pacucci C *et al*., Characterization of pomegranate (*Punica granatum* L.) genotypes collected in Puglia region, southeastern Italy. Sci Hortic 178:70–78 (2014).

[23] Passafiume R , Perrone A , Sortino G , Gianguzzi G , Saletta F , Gentile C *et al*., Chemical–physical characteristics, polyphenolic content and total antioxidant activity of three Italian‐grown pomegranate cultivars. NFS J 16:9–14 (2019).

[24] Zaouay F , Mena P , Garcia‐Viguera C and Mars M , Antioxidant activity and physico‐chemical properties of Tunisian grown pomegranate (*Punica granatum* L.) cultivars. Ind Crops Prod 40:81–89 (2012).

[25] Ampem G , Physico‐chemical and textural properties relevant to processing of pomegranate fruit and arils, in Quality Attributes of Pomegranate Fruit and Co‐products Relevant to Processing and Nutrition. Stellenbosch University, Sudafrica. Available: https://scholar.sun.ac.za, pp. 12–51 (2017).

[26] Fernandes L , Pereira JA , Lopez‐Cortes I , Salazar DM , Gonzalez‐Alvarez J and Ramalhosa E , Physicochemical composition and antioxidant activity of several pomegranate (*Punica granatum* L.) cultivars grown in Spain. Eur Food Res Technol 243:1799–1814 (2017).

[27] Adiletta G , Petriccione M , Liguori L , Pizzolongo F , Romano R and Di Matteo M , Study of pomological traits and physico‐chemical quality of pomegranate (*Punica granatum* L.) genotypes grown in Italy. Eur Food Res Technol 244:1427–1438 (2018).

[28] Zarei M , Azizi M and Bashir‐Sadr Z , Evaluation of physicochemical characteristics of pomegranate (*Punica granatum* L.) fruit during ripening. Fruits 66:121–129 (2011).

[29] Tehranifar A , Zarei M , Nemati Z , Esfandiyari B and Vazifeshenas MR , Investigation of physico‐chemical properties and antioxidant activity of twenty Iranian pomegranate (*Punica granatum* L.) cultivars. Sci Hortic 126:180–185 (2010).

[30] Chace EM , Church G and Poore H , The wonderful variety of pomegranate. USDA Circ 98:15 (1981).

[31] Ozgen M , Durgaç C , Serçe S and Kaya C , Chemical and antioxidant properties of pomegranate cultivars grown in the Mediterranean region of Turkey. Food Chem 111:703–706 (2008).

[32] Seeram N , Aviram M , Zhang Y , Henning S , Feng L , Dreher M *et al*., Comparison of antioxidant potency of commonly consumed polyphenol‐rich beverages in the United States. J Agric Food Chem 56:1415–1422 (2008). 18220345 10.1021/jf073035s

[33] Zhao X , Yuan Z , Fang Y , Yin Y and Feng L , Characterization and evaluation of major anthocyanins in pomegranate (*Punica granatum* L.) peel of different cultivars and their development phases. Eur Food Res Technol 236:109–117 (2013).

[34] Tapiero H , Tew KD , Nguyen Ba G and Mathé G , Polyphenols: do they play a role in the prevention of human pathologies? Biomed Pharmacother 56:200–207 (2002). 12109813 10.1016/s0753-3322(02)00178-6

[35] Kalaycıoğlu Z and Erim FB , Total phenolic contents, antioxidant activities, and bioactive ingredients of juices from pomegranate cultivars worldwide. Food Chem 221:496–507 (2017). 27979233 10.1016/j.foodchem.2016.10.084

[36] Qu W , Breksa AP III , Pan Z and Ma H , Quantitative determination of major polyphenol constituents in pomegranate products. Food Chem 132:1585–1591 (2012). 29243653 10.1016/j.foodchem.2011.11.106

