# Supplementary material for: Fresh pomegranate juices from cultivars and local ecotypes grown in southeastern Italy: comparison of physicochemical properties, antioxidant activity and bioactive compounds
Source: J Sci Food Agric. 2021 Aug 11;102(3):1185–92. doi: 10.1002/jsfa.11456 (PMC9291103; doi:10.1002/jsfa.11456)
Supplement: Supplementary file 1 — Appendix S1 Supporting information [file JSFA-102-1185-s001.docx]

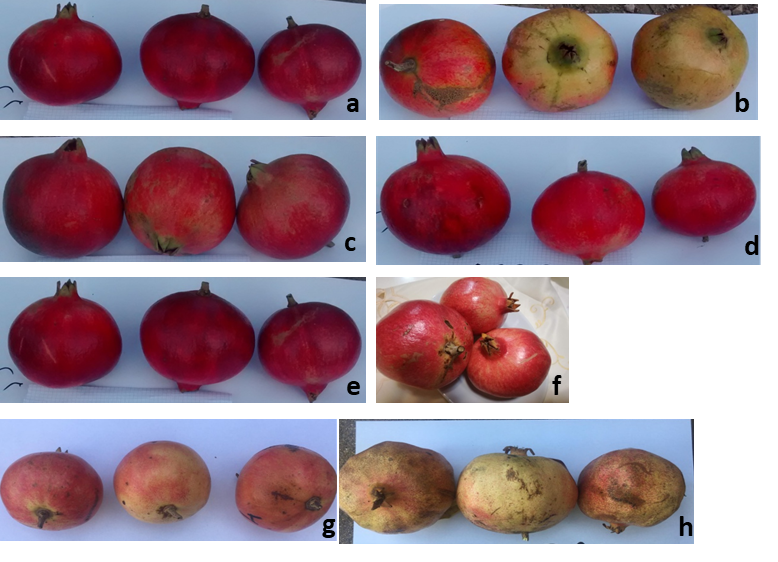


**Figure.** External appearance photographs of the different cultivars. a) Mollar; b) Jolly red; c) Dente di cavallo; d) Acco; e) Wonderful; f) Wonderful super; g) Eco FG; h) Eco BA
